# Supplementary material for: Peptidome Surveillance Across Evolving SARS-CoV-2 Lineages Reveals HLA Binding Conservation in Nucleocapsid Among Variants With Most Potential for T-Cell Epitope Loss in Spike
Source: Front Immunol. 2022 Jun 23;13:918928. doi: 10.3389/fimmu.2022.918928 (PMC9260595; doi:10.3389/fimmu.2022.918928)
Supplement: Supplementary file 1 [file DataSheet_1.pdf]

Supplementary Material for

Peptidome Surveillance Across Evolving SARS-CoV-2 Lineages Reveals HLA Binding Conservation in Nucleocapsid Among Variants With Most Potential for T-Cell Epitope Loss In Spike

Kamil Wnuk, Jeremi Sudol, Patricia Spilman, and Patrick Soon-Shiong

Supplementary Tables

Table S1. *Counts of immunogenic pMHC complexes with peptides originating from key SARS-CoV-2 proteins across 18 COVID-19 patients [15]*. From among all pMHC candidates evaluated, several were recognized by T cells across multiple patients. For all proteins except ORF3a, the majority of immunogenic peptides were from locations identified as pan-HLA hotspots.

Protein	Unique T-cell recognized pMHCs at pan-HLA hotspots	Unique T-cell recognized pMHCs	T-cell recognized pMHCs (across all patients)	pMHC candidates
S	14	18	18	616
N	4	4	7	177
ORF1ab	60	79	93	1868
ORF3a	1	11	13	140
M	5	6	6	138
E	1	1	1	28

Table S2. *ROC AUC and Mann-Whitney p-values to capture the separability of immunogenic vs. non-immunogenic pMHCs based on NetMHCpan-4.1 rank scores [15] and our RNN binding predictions*.

Protein(s)	NetMHCpan4.1 ROC AUC	RNN ROC AUC	RNN + hotspot ROC AUC	NetMHCpan4.1 Mann-Whitney p-value	RNN Mann-Whitney p-value	RNN + hotspot Mann-Whitney p-value
S + N	0.5406	0.5914	0.6366	0.258	0.076	0.018
All - ORF3a	0.5433	0.5337	0.5706	0.063	0.12	0.009
All	0.5503	0.529	0.5639	0.031	0.145	0.012
S	0.4838	0.5195	0.5833	0.408	0.392	0.126
N	0.7919	0.8439	0.839	0.023	0.01	0.013
ORF1ab	0.5185	0.5089	0.5434	0.289	0.395	0.105
ORF3a	0.6603	0.537	0.5179	0.039	0.344	0.428
M	0.3984	0.5242	0.5511	0.201	0.429	0.361
E	0.3333	0.1481	0.25	0.31	0.133	0.281

Supplementary Figures

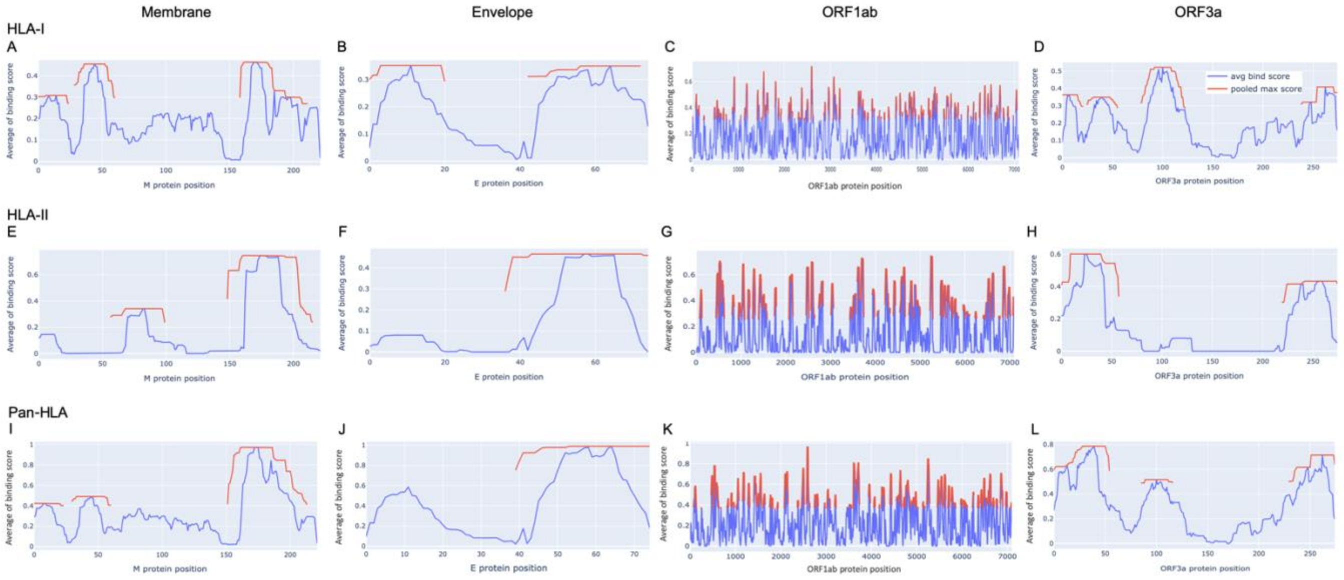

Figure S1. *Epitope hotspots in four additional SARS-CoV-2 proteins*. Protein regions with peak frequency of predicted binding peptides (potential epitope hotspots) across HLAs are indicated in red for 4 additional proteins from the SARS-CoV-2 reference genome (NCBI Reference Sequence: NC_045512): Membrane (A,E,I), Envelope (B,F,J), Open Reading Frame 1ab (ORF1ab) (C,G,K), and ORF3a (D,H,L). Red lines show the value of the nearest maxima of the aggregate signal (blue) within a set sliding window size (9 amino acids for HLA-I, 15 for HLA-II, 12 for pan-HLA). For each protein we show hotspots on aggregate signals across all HLA-I molecules only (A-D), HLA-II only (E-H), as well as the combined pan-HLA signal (I-L). The legend in panel D applies to all panels.

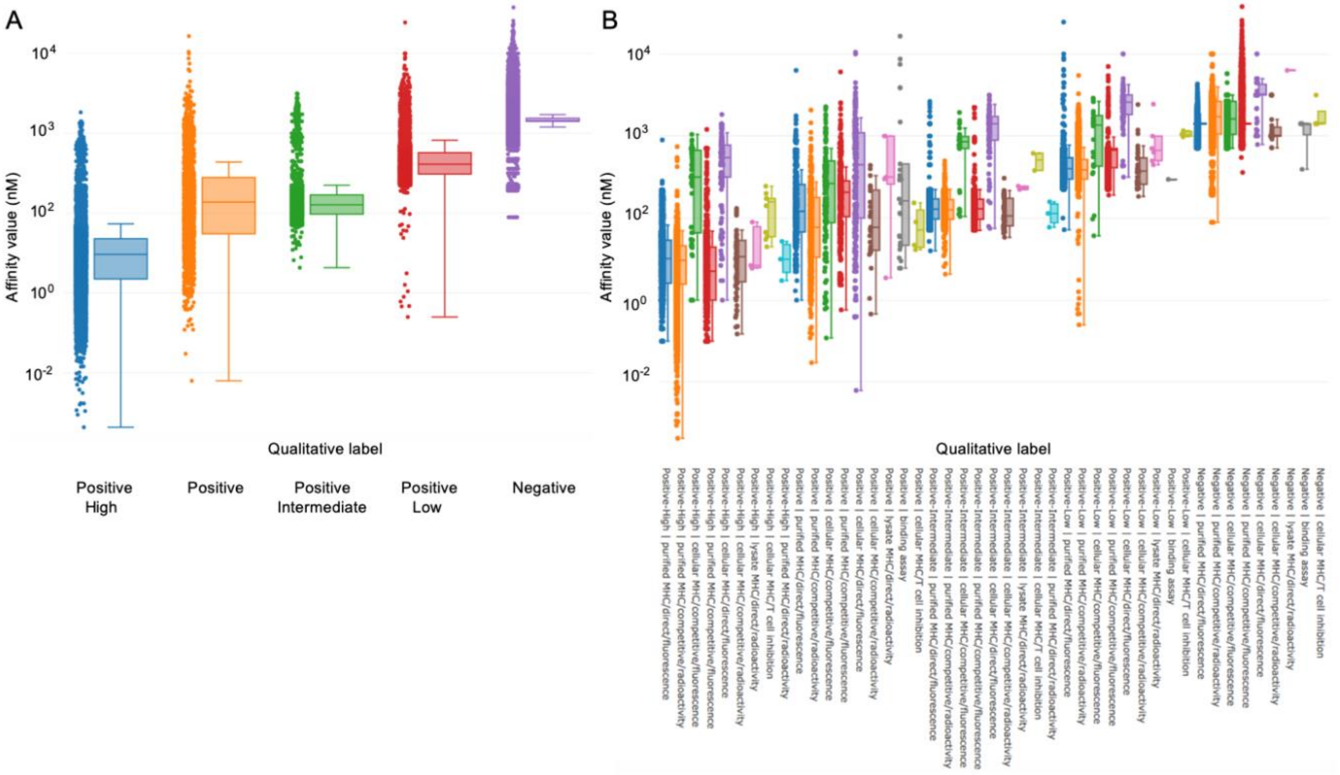

Figure S2. *IEDB affinity measurements*. (A) IEDB affinity measurements are represented on the y-axis and curated qualitative assessments of binding on the x-axis. (B) Affinity is shown on the y-axis and a qualitative label presented by “assay/method” used to measure affinity on the x-axis. Each assay or method was assigned a distinct color.

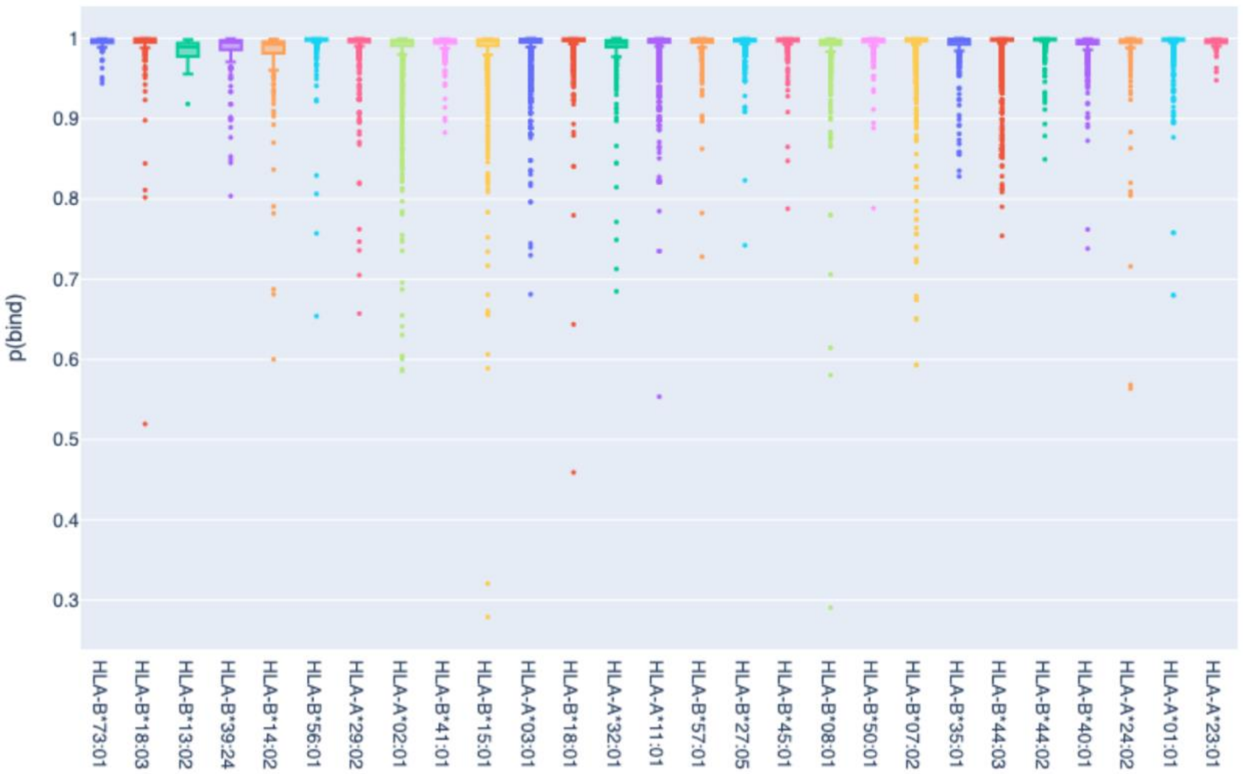

Figure S3. *Box plots of all RNN classifier binding predictions across 27 HLA-I molecules*. Empirically verified eluted HLA-I ligands in the Pearson *et al.* dataset [36] are shown in the x-axis and our binding prediction values on the y-axis.

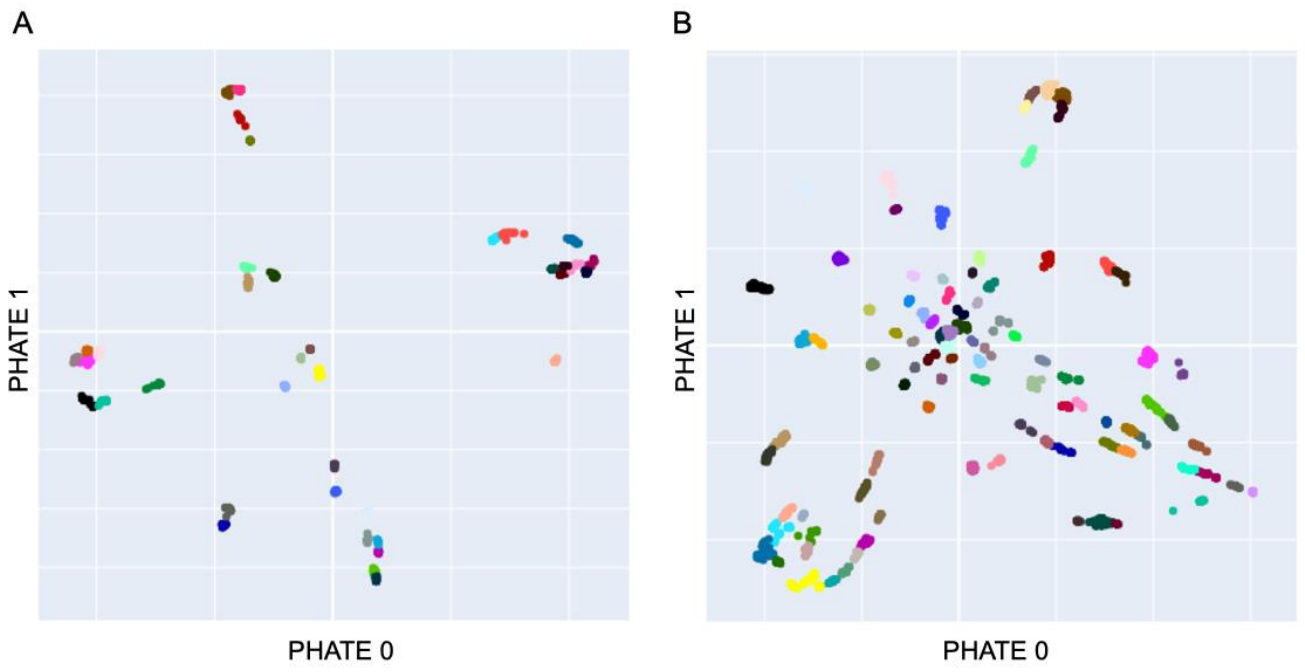

Figure S4. *PHATE* [46] *visualization of HLA-I and HLA-II molecules*. (A) Visualization of clustered RNN embeddings of all HLA-I A, B, and C molecules for which full amino acid sequences were available. Agglomerative clustering was used to group embeddings into 38 clusters, illustrated in the plot as discrete colors. (B) PHATE visualization and clustering of all permutations of compatible HLA-II alpha and beta chain embeddings, with a stable and silhouette score optimal point at 93 clusters.

Interactive visualizations

Interactive visualizations allowing researchers to delve deeper into our data and findings are available at: https://research.immunitybio.com/scov2_epitopes/

Supplementary data

Data Sheet 2: RNN_vs_CNN_vs_NetMHC41_perHLA_results.csv *Per-HLA classification metrics used to report mean results in Table 2*. The table summarizes the sample count ("n_pos", "n_neg"), how many samples were classified as ambiguous by each system ("RNN_n_ambig", "CNN_n_ambig"), and the fraction of the total sample count classified as ambiguous ("RNN_n_ambig_fract", "CNN_n_ambig_fract"). Data reported from competing systems for individual HLAs was obtained from Reynisson et al. [34] Supplementary Table 8. Additionally, we report PR AUC for each HLA, which was not reported across the systems evaluated in [34].

Data Sheet 3: rnn_results.zip. *RNN predictions for all peptides in benchmark test data.* The archive contains 36 csv files that include the means and standard deviations of our model ensembles, as well as classification labels of {'binder', 'not-binder', 'ambiguous'}. The test data used were available online from the authors of NetMHCPan4.1 [34]: https://services.healthtech.dtu.dk/service.php?NetMHCpan-4.1

Data Sheet 4: cnn_results.zip. *CNN predictions for all peptides in benchmark test data.* The archive contains 36 csv files that include the means and standard deviations of our model ensembles, as well as classification labels of {'binder', 'not-binder', 'ambiguous'}. The test data used were available online from the authors of NetMHCPan4.1 [34].

Data Sheet 5: hla1_clusters_and_selection.csv. *HLA-I cluster label assignment and selection.* Cluster label assignments for all processed HLA-I, as well as those selected for our analysis set indicated in column "selected".

Data Sheet 6: hla2_ab_clusters_and_selection.csv. *HLA-II cluster label assignment and selection.* Cluster label assignments for all processed HLA-II, as well as those selected for our analysis set indicated in column "selected_ab".
